# Anti-inflammatory Effects of *S. cumini* Seed Extract on Gelatinase-B (MMP-9) Regulation against Hyperglycemic Cardiomyocyte Stress

**DOI:** 10.1155/2021/8839479

**Published:** 2021-03-03

**Authors:** Neha Atale, Chandra Bhushan Mishra, Shrey Kohli, Raj Kumar Mongre, Amresh Prakash, Sweta Kumari, Umesh C. S. Yadav, Raok Jeon, Vibha Rani

**Affiliations:** ^1^Department of Biotechnology, Jaypee Institute of Information Technology, A-10, Sector-62, Noida, 201307 Uttar Pradesh, India; ^2^College of Pharmacy, Sookmyung Women's University, Hyochangwon-gil 52, Yongsan-13 Gu, Seoul 140-742, Republic of Korea; ^3^Institute for Laboratory Medicine, Clinical Chemistry and Molecular Diagnostics, University of Leipzig (Medical Faculty), Leibigstr. 27, 04103 Leipzig, Germany; ^4^Amity Institute of Integrative Sciences and Health, Amity University, Gurgaon 122413, Haryana, India; ^5^Division of Biochemistry, Indian Agricultural Research Institute (IARI), Pusa Campus, 110012, New Delhi, India; ^6^School of Life Sciences, Central University of Gujarat, Sector-30, Gandhinagar, 382030 Gujarat, India

## Abstract

Black berry (*Syzygium cumini*) fruit is useful in curing diabetic complications; however, its role in diabetes-induced cardiomyopathy is not yet known. In this study, we investigated the regulation of gelatinase-B (MMP-9) by *S. cumini* methanol seed extract (MSE) in diabetic cardiomyopathy using real-time PCR, RT-PCR, immunocytochemistry, gel diffusion assay, and substrate zymography. The regulatory effects of MSE on NF-*κ*B, TNF-*α*, and IL-6 were also examined. Identification and estimation of polyphenol constituents present in *S. cumini* extract were carried out using reverse-phase HPLC. Further, *in silico* docking studies of identified polyphenols with gelatinase-B were performed to elucidate molecular level interaction in the active site of gelatinase-B. Docking studies showed strong interaction of *S. cumini* polyphenols with gelatinase-B. Our findings indicate that MSE significantly suppresses gelatinase-B expression and activity in high-glucose- (HG-) stimulated cardiomyopathy. Further, HG-induced activation of NF-*κ*B, TNF-*α*, and IL-6 was also remarkably reduced by MSE. Our results suggest that *S. cumini* MSE may be useful as an effective functional food and dietary supplement to regulate HG-induced cardiac stress through gelatinase.

## 1. Introduction

*Syzygium cumini*, a seasonal perishable berry, commonly known as malabar plum, belongs to the family Myrtaceae. The plant is native to Asia and Oceanic regions, mainly India, China, and New Zealand. It is also grown in East Africa, South America, and tropical parts of the USA [1, 2]. The purple fruits of jamun are used for the processing of chips, vinegar, jams, smoothies, and squashes and hold a significant position in the functional food industry. Besides the fruits, other parts of the plant also have been found useful in treating chronic diseases including diabetes-related complications [3, 4]. The plant of *S. cumini*, especially its fruit, is considered a functional food, as it consists of plenty of polyphenols such as gallic acid, quercetin, *β*-sitosterol, eicosane, diphenic acid, ellagic acid, isoquercetin, and myricetin, which may facilitate healthy benefits against diabetes-induced detrimental changes and also reduce the risk of neurological and cardiovascular diseases (CVDs) [5]. These molecules are known for their anti-inflammatory, antihyperlipidemic, antioxidative, free radical scavenging, and antidiabetic potential [6]. Further studies have shown that *S. cumini* seed extracts function as a chemopreventive agent against DNA damages and also have antimutagenic as well as antigenotoxic effects [7].

Cardiac stress has become a leading cause of morbidity and mortality among people with type 2 diabetes. Diabetic individuals have two to four times greater risks of cardiac arrest than nondiabetic people [8, 9]. The alarming rate of diabetes incidences and its impact on the heart make diabetic cardiomyopathy a challenging health condition worldwide. However, the effect of *S. cumini* seed polyphenolic constituents on diabetes-induced cardiac stress is not known. Thus, it is important to study the effect of MSE against diabetes-induced cardiac stress along with its mechanism of action which may open a new way for the management of cardiomyopathy.

Hyperglycemia is known to cause cardiac stress, which eventually leads to cardiomyopathy through increased activity of matrix metalloproteinases (MMPs) and extracellular matrix (ECM) remodeling [10]. MMPs are a family of secreted and membrane-bound zinc and calcium-dependent endopeptidases, which include collagenases, gelatinases, stromelysins, matrilysins, and a few peptidases [11–13]. The variation in the expression of MMPs and endogenous tissue inhibitors of matrix metalloproteinases (TIMPs), especially TIMP-1 and TIMP-2, may lead to the pathophysiology of various diseases including cardiovascular diseases, cancer, diabetes, and neurodegenerative disorders [14]. TIMPs act as inhibitors for active MMPs by binding to the Zn^2^+ in its catalytic domain [14, 15]. Their production is regulated by a variety of mediators including cytokines, chemokines, hormones, and growth factors [16]. The inflammatory cytokines and growth factors released at tissue damage sites lead to the enhancement of the expression of MMP-9 and among which TNF-*α* is found to be a potent stimulator of MMP-9 transcription and atherosclerosis [17].

The central role of MMPs in ECM remodeling, therefore, makes them a potential drug target in diabetic cardiomyopathy [18, 19]. One of the critical MMPs known to be activated in diabetes is MMP-9, the largest and the most complex member which otherwise remains latent in healthy hearts. During diabetic cardiomyopathies, gelatinase-B also called MMP-9 activated via Ras/Raf/MEK/ERK signaling cascade causes extensive degradation of ECM and decreases matrix turnover, which is associated with several cardiac abnormalities and heart failure [20]. Although there has been a big thrust towards the development of synthetic MMP inhibitors, their safe implementation and testing in successful clinical trials is still a challenge [21].

Overwhelming evidence from epidemiological, *in vivo*, *in vitro*, and clinical studies indicates that plant-based functional foods not only provide basic nutrition but also have the potential to suppress diseases and ensure good health and longevity. Therefore, efforts to identify new plant-based MMP inhibitors, with less toxicity and more specificity, are required [22]. To date, specific studies revealed the inhibition of MMPs by several natural products such as dietary gallic acid, epigallocatechins, anthocyanins, curcumin, and caffeic acid in various pathological conditions [23, 24].

*Syzygium cumini* has been extensively studied for its nutrient composition and found to be enriched with polyphenols and could serve as a source of a potential MMP inhibitor that can be effective in HG-induced cardiac stress [6, 25]. Our previous studies found that *S. cumini* MSE significantly suppressed the HG-induced stress in H9C2 cardiomyocytes by reducing mitochondrial membrane potential, reactive oxygen species (ROS) overproduction, and collagen levels, suggesting its potential as a functional food and supplementary diet to diabetic patients for suppression of cardiac stress [26]. In the present study, we have examined the role of MSE as well as the purified component of seed extract on MMP-9 expression and activity in HG-stressed cardiomyocytes. Our current study also proposes *S. cumini* to be a potent MMP inhibitor and a therapeutic agent in diabetes-induced cardiac complications.

## 2. Materials and Methods

A schematic representation of the methodology used in the present study is illustrated in Fig. S1.

### 2.1. Chemicals

All chemicals were purchased from Sigma Aldrich (St. Louis, MO, USA). All the antibodies were purchased from Santa Cruz Biotechnology Inc. (Dallas, TX, USA).

### 2.2. Seed Collection

*S. cumini* seeds were procured from a local vendor in the month of July-August and were authenticated by Dr. Anshu Rani, an eminent botanist, Department of Botany, Govt. P.G. College, Abu Road, Rajasthan, India. Further, the seeds were washed, ground, and dried.

### 2.3. Preparation of Methanol Extract of *S. cumini* Seeds

The methanol extract of *S. cumini* seeds (MSE) was prepared by using the Soxhlet solvent extraction method. Seed powder (20 g) was mixed with 200 ml methanol. The temperature was set at its boiling point (64.7°C), and 12-14 cycles were run for complete extraction. After solvent evaporation in a rotary evaporator, the dried powder was reconstituted at the concentration of 1 mg/ml in molecular-grade water.

### 2.4. Cell Culture

The rat heart-derived H9C2 cardiomyoblast cells were obtained from the National Centre for Cell Science (NCCS), Pune, India (originally from ATCC, USA). H9C2 cells were cultured with Dulbecco's modified Eagle's medium (DMEM) supplemented with penicillin (100 unit/ml), streptomycin (100 *μ*g/ml), glucose (5.5 mM), L-glutamine (2 mmol/l), sodium bicarbonate (3.7 g/l), 10% fetal bovine serum (FBS), insulin (50 mg/ml), transferrin (27.5 mg/ml), selenium (0.025 mg/ml), and amphotericin B (5 *μ*g/ml) in a humidified CO_2_ incubator (New Brunswick Scientific, NJ, USA) with 5% CO_2_ at 37°C [27].

### 2.5. Treatment of Cells with Glucose, *S. cumini* MSE, and Gallic Acid (GAL)

H9C2 cells were induced with optimized doses of glucose and *S. cumini* MSE as standardized in our previous studies [28]. The gallic acid was used as a purified equivalent in all the experiments. Our study included six groups: (a) control (untreated) cells, (b) cells induced with 4.5 mg/ml glucose (HG), (c) high-glucose-induced cells treated with *S. cumini* MSE (9 *μ*g/ml) (HG+MSE), (d) MSE-treated cells (MSE), (e) high-glucose-induced cells treated with gallic acid (3.4 *μ*g/ml) (HG+GAL), and (f) gallic acid-treated cells (GAL).

### 2.6. Cell Viability Assay

Cell viability was determined by a 3-(4,5-dimethyl-thiazol-2-yl)-2,5-diphenyl tetrazolium bromide (MTT) assay (Ferrari, Fornasiero & Isetta, 1990). Approximately 8 × 10^3^ H9C2 cells were seeded in 96-well plates. The H9C2 cells were treated as described above and incubated for 48 h at 37°C in a humidified incubator containing 5% CO_2_. After completion of incubation, 10 *μ*l MTT (5 mg/ml) was added, and cells were further incubated for 3 h. The culture supernatant was then discarded, and formazan crystals were dissolved in 100 *μ*l DMSO. Absorbance was measured through an ELISA plate reader (ThermoFisher Scientific Inc., Waltham, MA, USA) at 570 nm. Cell viability was defined in relation to control cells as the ratio of absorbance of the treated sample to absorbance of the control sample.

### 2.7. Haematoxylin-Eosin (H&E) Staining

The H&E staining was performed for the determination of morphology of cells. H9C2 cells were seeded in six-well plates as per the experimental sets described earlier and incubated for 48 h. Subsequently, cells were washed with cold phosphate-buffered saline (PBS) and fixed with 100% chilled (-20°C) methanol. Haematoxylin (0.5% alcoholic solution) was added and incubated for 30 min at 25°C. After washing twice with PBS at room temperature, cells were counterstained with 5% eosin, washed, and mounted. The cells were observed under a light microscope, and images were captured at 40x magnification. The stained cells were eluted with 0.1 N NaOH, and the absorbance of samples was measured at 560 nm.

### 2.8. Extraction of Total Cell Protein

After completion of 48 h of incubation, cells were harvested and washed with cold PBS. The cell pellet was lysed in precooled 1x RIPA buffer (50 mM Tris-HCl (pH 7.5), 150 mM NaCl, 500 mM Na_2_EDTA, 1 mM EGTA, 1% NP-40, 1% sodium deoxycholate, 2.5 mM sodium pyrophosphate, and protease inhibitor cocktail (104 mM AEBSF, 80 *μ*M aprotinin, 4 mM bestatin, 1.4 mM E-64, 2 mM leupeptin, and 1.5 mM pepstatin A) on ice for 1 h. The cell lysate was centrifuged at 13000 × g for 15 min at 4°C in a refrigerated centrifuge. The concentration of total cell proteins in supernatant was estimated by a Bradford assay.

### 2.9. Gel Diffusion Assay

A solution containing 1.5% agarose, digestion buffer (50 mM Tris-Cl (pH 7.4), 150 mM NaCl, 5 mM CaCl_2_, and 0.02% Brij-45), and 1 mg/ml gelatin was poured on a gel plate and allowed to solidify. An equal amount of protein (30 *μ*g/ml) from various experimental sets were loaded into wells punched in the solidified agarose gel and incubated overnight at 37°C. Zones of gelatin digestion were detected by staining the agarose gel in a solution containing 0.25% Coomassie Brilliant Blue R-250. The enzymatic activity was estimated as a function of the diameter of the digested zone compared to standard trypsin.

### 2.10. Zymographic Analysis of MMP-9 Production

The equal amount of protein (30 *μ*g/ml) from different experimental sets was mixed with 2x sample buffer (0.005% Bromophenol Blue, 20% glycerol, 4% SDS, 100 mM Tris-Cl (pH 6.8)) in equal proportion and subjected to electrophoresis in 10% polyacrylamide gel containing gelatin (1 mg/ml). After washing with 2.5% Triton X-100, the gels were incubated in digestion buffer, stained with 0.25% Coomassie Brilliant Blue R-250, and further destained to visualize gelatinolytic activities in zymogram which was observed as transparent bands against the background of Coomassie Blue-stained gelatin containing gel. The quantitation was done using NIH ImageJ software.

### 2.11. Cell *In Situ* Zymography

H9C2 cells were cultured on coverslips and were fixed with chilled (-20°C) methanol and implanted in the uniformly spread mixture of 0.5% agarose containing 0.1% fluorescein-conjugated gelatin on the glass slides. The cells were incubated at 37°C for 1 h in developing buffer (50 mM Tris-Cl (pH 7.4), 150 mM NaCl, 5 mM CaCl_2_, 0.02% Brij-45) and visualized under a fluorescent microscope (Olympus Corporation, TYO, Japan). Images were captured at 40x magnification.

### 2.12. Immunocytochemistry

H9C2 cells were grown on coverslips under different experimental conditions and fixed with methanol. After washing the cells with PBS, blocking was done for 1 h at room temperature (25°C) using 3% BSA prepared in PBS followed by incubation with primary antibodies (against MMP-9, NF-*κ*B, GAPDH, and Lamin A/C) for 1 h at 37°C. Subsequently, cells were stained with FITC-conjugated secondary anti-goat antibody under similar conditions. Nuclei were counterstained with 4′,6-diamidino-2-phenylindole (DAPI) as described previously [29] and observed under a fluorescent microscope (Olympus Corporation, TYO, Japan). Images were captured using a Progress capture camera fitted to the microscope. Overlay images were obtained by NIH ImageJ software.

### 2.13. RT-PCR

After completion of incubation, total RNA was extracted using a TRIzol reagent (Invitrogen Carlsbad, CA, USA) as per the manufacturer's manual. Reverse transcription of all sets with 0.1 *μ*g total RNA was performed using a RevertAid H Minus First Strand cDNA Synthesis Kit (ThermoFisher Scientific Inc., Waltham, MA, USA) as per instructions provided. The cDNA samples were diluted to 20 ng/*μ*l. The thermal cycling conditions were composed of an initial denaturation step at 95°C for 5 min followed by 30 cycles at 95°C for 30 s each, the respective annealing temperature for 30 s and 72°C for 30 s, and a final extension of 10 min at 72°C. Amplified products were resolved on 2% agarose gels and visualized by ethidium bromide staining using GelDoc (BioRad Laboratories, CA, USA). The primer sequences for specific genes and their respective annealing temperatures were as follows: MMP-9: 5′-CACCGCTCACCTTCACCCG-3′ (F), 5′-TGCCGAGTTGCCCCCAGTTA-3′ (R), 66°C; TNF-*α*: 5′-AGAAAGTCAGCCTCCTCTCC-3′ (F), 5′-ACTCCAAAGTAGACCTGCCC-3′ (R), 56°C; IL-6: 5′CCTACCCCAACTTCCAATGCTC-3′ (F), 5′-TTGGATGGTCTTGGTCCTTAGCC-3′ (R), 58.5°C; and *β*-Actin: 5′-CATCGTACTCCTGCTTGCTG-3′ (F), 5′-CCTCTATGCCAACACAGTGC-3′ (R), 57.5°C. To further validate the semiquantitative results, qRT-PCR was carried out using SYBR Green PCR master mix of the PikoReal™ Real-Time PCR System (ThermoFisher Scientific Inc. Waltham, MA, *USA*). Upon completion, fold changes in gene expression were calculated by a delta delta Ct (∆∆Ct) method. The fold change in gene expression was normalized to an internal *β*-actin control gene.

### 2.14. HPLC Analysis

Methanol extract of *S. cumini* seeds and 10 standards, namely, ellagic acid, gallic acid, p-coumaric acid, quercetin, protocatechuic acid, sinapic acid, caffeic acid, kaempferol, ferulic acid, and p-hydroxybenzoic acid (Sigma-Aldrich, St. Louis, MO, USA), was subjected to HPLC analysis by using Shimadzu LC instrument equipped with Hypersil C-18 column and PDA detector (Shimadzu Corporation, Kyoto, Japan). Mobile phase containing methanol/acetonitrile (98/2 *v*/*v*) was used to perform HPLC analysis [30–32]. The flow rate was set at 0.5 ml/min, and the absorbance used for this study was 254 nm. 100 *μ*l MSE, taken from 150 ml stock solution obtained from 10 g *S. cumini* seed powder, was diluted with 900 *μ*l HPLC-grade methanol and filtered through 0.2 *μ* filter (Millipore, MA, USA). From the diluted stock, 20 *μ*l was used for HPLC analysis. For HPLC analysis of the standards, 1 mg/ml stocks were prepared in methanol, and 20 *μ*l of each standard was subjected to HPLC analysis. The concentration of each component of the extract was calculated by using the area of different standards individually. The results are represented as the mean ± SD, and each experiment is performed in triplicate.

### 2.15. Molecular Docking

AutoDock Vina was used for docking studies [33]. The X-ray crystallographic structure of MMP-9 (Pdb Id 4H82) was selected from the protein structure database (protein data bank RCSB PDB). The ligand molecular structure of ellagic acid, gallic acid, p-coumaric acid, quercetin, protocatechuic acid, sinapic acid, caffeic acid, kaempferol, ferulic acid, and p-hydroxybenzoic acid was designed and 3D optimized using ACD ChemSketch 12.0. A grid box of 20 × 20 × 20 points was generated around the active site coordinates of MMP-9 protein. The binding free energies of the ligand-protein interactions were also calculated using the best-predicted conformations in the bounded state and compared with binding free energies of MMP-9-doxycycline interactions [34–39].

### 2.16. Statistical Analysis

The data are expressed as means ± SD. Statistical analysis was performed using SPSS 16 software. Statistical comparisons were made with one-way ANOVA with Tukey's test. All the results were considered significant with a *p* value ≤ 0.05. Each experiment was conducted in triplicates and repeated thrice.

## 3. Results

### 3.1. *S. cumini* Prevents Glucose-Induced Cardiac Stress

**Figure 1:**
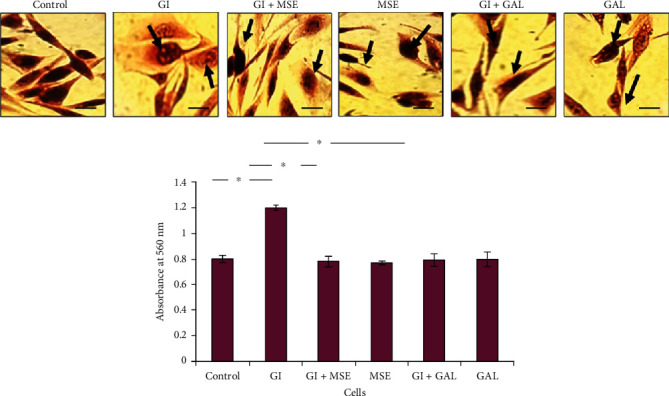
Morphological analysis in H9C2 cells by haematoxylin-eosin (H&E) staining. Light-field micrographs (40x magnifications) showing an increase in cell size in high-glucose- (HG-) induced H9C2 cells, and *S. cumini* methanol seed extract (MSE) treatment prevented such increase as indicated by arrows. Gallic acid treatment also showed decreased cell size near the control. Quantitation of cell size was represented by histogram (^∗^*p* ≤ 0.05). Scale bar: 20 *μ*.

To evaluate the protective effect of *S. cumini* on glucose-induced cardiac stress, H9C2 cardiomyocytes were treated with high glucose (4.5 mg/ml, 25 mM) alone or high glucose concomitantly with *S. cumini* MSE. Gallic acid which is a known cardioprotective agent under diabetes conditions was used as a positive control. Morphological assessment by H&E staining showed that although high-glucose treatment results in about 1.5-fold increase in cell size, treatment with MSE prevented the increase in cell size as effective as gallic acid treatment (Figure 1). Furthermore, neither MSE nor gallic acid resulted in any cytotoxic or morphological alterations indicating that either treatment does not exert any harmful effects on cardiac cells themselves but are protective under glucose-induced stress conditions.

### 3.2. *S. cumini* Prevents Glucose-Induced Gelatinase Activity in H9C2 Cardiomyocytes

**Figure 2:**
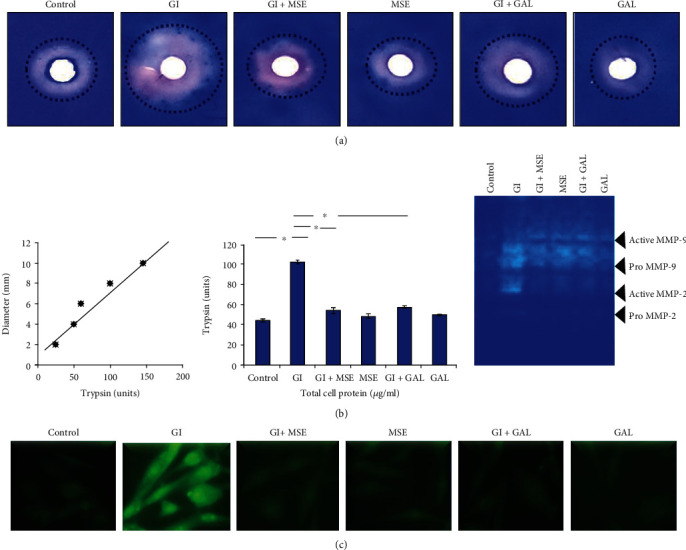
Effect of *S. cumini* methanol seed extract (MSE) on gelatinolytic activity. (a) Gel-diffusion assay: gelatinolytic activity was measured by digested zones of gelatin around the wells. Standard curve of trypsin was plotted (enzyme units), and enzyme activity for the samples was calculated from the standard graph of trypsin and represented as histogram. The width of the digested zone is proportional to the extent of substrate cleavage and can be used to quantitate protease activity. After giving treatment, we compared our results with trypsin digested zones and found the gelatinase activity in trypsin units. (b) Gelatin zymography: to depict the MSE effect on matrix metalloproteinase (MMP) enzyme activity. Lane 1: control; Lane 2: glucose induced (GI); Lane 3: glucose induced+*S. cumini* methanol seed extract (GI+MSE) treated; Lane 4: MSE-treated alone (MSE); Lane 5: glucose induced+gallic acid treated (GI+GAL); Lane 6: gallic acid alone (GAL) (^∗^*p* ≤ 0.05). (c) Cell *in situ* zymography: fluorescence micrographs showing the increase in gelatinolytic activity in HG-induced cells whereas *S. cumini* methanol seed extract (MSE) and gallic acid (GAL) treatment of HG-glucose stressed cells reverses the activity near the control. Images captured at 40x magnification (^∗^*p* ≤ 0.05). Scale bar 20 *μ*m.

Cardiac remodeling involving molecular changes that manifest as increased cell size under disease conditions is known to be regulated by ECM remodeling matrix metalloproteinases (MMPs 2& 9), popularly known as gelatinases. We, therefore, studied whether the protective effect of *S. cumini* MSE is regulated by these gelatinases. We first studied the ability of lysates from H9C2 cardiomyocytes to digest gelatin as a substrate in a gel-diffusion assay evaluated by the area of the digestion zone (Figure 2(a)). We found that treatment with high glucose resulted in an enhanced gelatinase activity (equivalent to 102 U trypsin) compared to untreated controls (equivalent to 45 U trypsin) which was prevented upon concomitant treatment with *S. cumini* MSE (equivalent to 55 U trypsin). Likewise, gallic acid was also protective against glucose-induced gelatinase activity (equivalent to 58 U trypsin), and gallic acid or MSE treatment alone did not result in any significant change in gelatinase activity (equivalent to 49 U and 50 U trypsin, respectively) compared to controls. We then performed gelatin zymography using protein lysates under similar experimental conditions to evaluate the MMP activity (Figure 2(b)). We found similar results as obtained above where high-glucose treatment resulted in enhanced MMP activity which was prevented by concomitant treatment with *S. cumini* MSE or gallic acid. In order to further confirm the protective effects of *S. cumini* MSE on glucose-induced MMP activity, we then performed cell *in situ* zymography to map the in-position cell matrix metalloproteinase activity using FITC-conjugated gelatin (Figure 2(c)). We found that cells treated with high glucose alone were much more efficient in digesting FITC-gelatin resulting in enhanced fluorescence intensity compared to controls indicating an increased MMP activity. Intriguingly, treatment with either *S. cumini* MSE or gallic acid under high-glucose conditions resulted in only a weak fluorescence signal indicating a reduced MMP activity, and either treatment was protective. Akin to the results obtained above, MSE or gallic acid treatment alone did not have any significant effect on MMP activity compared to the control. Overall, these results suggested that *S. cumini* MSE exerts an inhibitory effect on glucose-induced MMP activity in cardiomyocytes which was as competent as gallic acid, a previously known cardioprotective agent as well as a phytocomponent of *S. cumini*.

### 3.3. *S. cumini* Prevents Glucose-Induced Gelatinase-B Expression in H9C2 Cardiomyocytes

**Figure 3:**
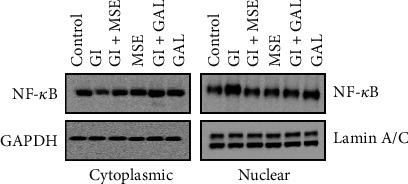
Expression analysis of gelatinase-B. (a) RT-PCR studies: Lane 1: control; Lane 2: glucose induced (GI); Lane 3: glucose induced+*S. cumini* methanol seed extract (GI+MSE) treated; Lane 4: MSE-treated alone (MSE); Lane 5: glucose induced+gallic acid treated (GI+GAL); Lane 6: gallic acid alone (GAL) (^∗^*p* ≤ 0.05). Expression levels of MMP-9 were obtained by qRT-PCR and normalized against *β*-actin. Fold changes were shown as histogram (^∗^*p* ≤ 0.05). (b) Immunocytochemistry for gelatinase-B: photomicrographs showing expression of MMP-9 in different experimental groups at 20x magnification. Scale bar 10 *μ*m.

Although a generalized MMP activation in cardiomyopathies has been witnessed, a specific induction of gelatinase-B has been reported in diabetic cardiomyopathy. We therefore selected gelatinase-B as a potential therapeutic target for further assessment. Semiquantitative RT-PCR as well as qRT-PCR analysis showed that high-glucose treatment results in an elevated mRNA expression of gelatinase-B compared to the control (Figure 3(a)). Furthermore, treatment with *S. cumini* MSE under high-glucose conditions significantly prevented the increase in gelatinase-B mRNA expression. Intriguingly, this inhibitory effect of *S. cumini* MSE on gelatinase-B mRNA expression was stronger as compared to that of gallic acid. Immunohistochemistry analysis of H9C2 cardiomyocytes treated under similar conditions showed that *S. cumini* MSE treatment under high-glucose conditions prevented the increase in gelatinase-B protein expression (Figure 3(b)). Taken together, these results suggested that *S. cumini* MSE suppresses the gelatinase-B protein as well as mRNA expression in high-glucose-treated H9C2 cardiomyocytes.

### 3.4. *S. cumini* Prevents Glucose-Induced NF-*κ*B Nuclear Translocation and Cardiac Inflammation

**Figure 4:**
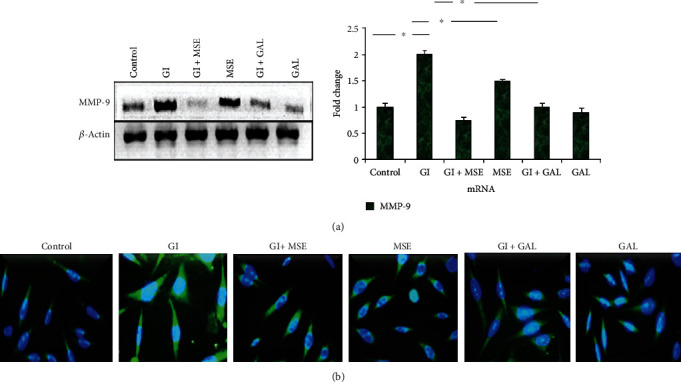
Nuclear factor-*κ*B (NF-*κ*B) localization in H9C2 cells. Western blotting to observe localization of NF-*κ*B from cytoplasm to nucleus using p65 antibody: nuclear localization of NF-*κ*B (green fluorescence in nuclei) in high-glucose- (HG-) induced cells. DAPI staining showed nuclear morphology. GAPDH and Lamin A/C were used as controls for cytoplasmic and nuclear extracts, respectively.

MMP expression has been shown to be regulated by redox-sensitive transcription factor NF-*κ*B. Importantly, we observed that *S. cumini* treatment altered not only gelatinase-B protein expression but also its mRNA expression. This indicated that the protective effect of *S. cumini* on gelatinase-B could be regulated at the transcriptional level. NF-*κ*B transcriptional activity is dependent upon its translocation to the nucleus. Therefore, we studied the nuclear localization of NF-*κ*B protein using antibodies of p65 (RelA) subunit of NF-*κ*B protein by immunoblotting (Figure 4). We observed that upon high-glucose treatment, NF-*κ*B translocated more from the cytoplasm to the nucleus compared to controls indicating its activation. However, treatment of *S. cumini* along with high glucose shows a reduced localization of NF-*κ*B in the nucleus compared to the cytoplasm and was similar to that observed in controls. Similarly, gallic acid treatment under high glucose conditions also showed its predominant localization in the cytoplasm indicating its reduced activation. These results suggested that *S. cumini* MSE treatment inhibits NF-*κ*B nuclear translocation further controlling their gelatinase-B expression.

**Figure 5:**
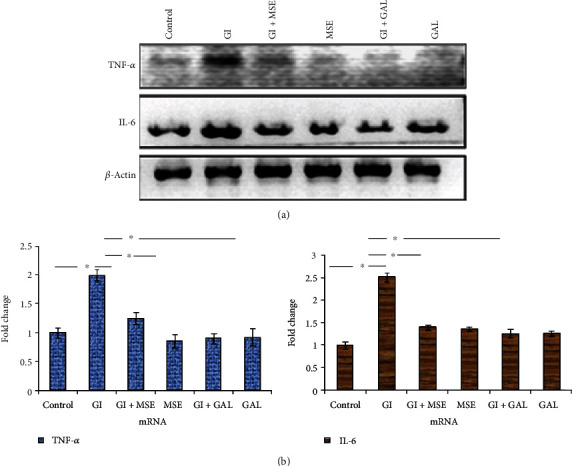
RT-PCR profiles of tumor necrosis factor-*α* (TNF-*α*) and interleukin-6 (IL-6) in different experimental groups. Lane 1: control; Lane 2: glucose induced (GI); Lane 3: glucose induced+*S. cumini* methanol seed extract (GI+MSE) treated; Lane 4: MSE-treated alone (MSE); Lane 5: glucose induced+gallic acid treated (GI+GAL); Lane 6: gallic acid alone (GAL). (a) Semiquantitative PCR showed significant increase in expression for both the markers in the cells treated with high glucose (HG). *S. cumini* methanol seed extract (MSE) and gallic acid (GAL) treatment reduced it remarkably up to the control. (b) Results obtained by qRT-PCR (real-time PCR) were normalized against *β*-actin and represented by histogram (^∗^*p* ≤ 0.05).

An increase in NF-*κ*B nuclear translocation would enhance not only gelatinase-B expression but other proinflammatory cytokines such as TNF-*α* and IL-6. We, therefore, evaluated the mRNA expression of these cytokines (Figures 5(a) and 5(b)). We found that high-glucose treatment exacerbated the expression of TNF-*α* and IL-6, respectively, and *S. cumini* MSE treatment was able to prevent it. Gallic acid treatment also decreased the expression of TNF-*α* and IL-6 similar to *S. cumini* MSE. On the one hand, these results suggested that *S. cumini* MSE is able to prevent glucose-induced cardiac inflammation; on the other hand, it corroborated a reduced activity of NF-*κ*B. However, additional evidence would be required to assert a direct correlation between NF-*κ*B activity and cytokine expression.

### 3.5. *S. cumini* Phenolic Compounds Block Gelatinase-B Substrate Binding

**Figure 6:**
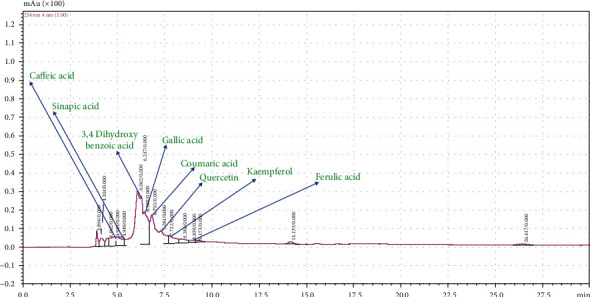
High-performance liquid chromatography (HPLC) analysis of *S. cumini* methanol seed extract (MSE). HPLC chromatogram to depict various peaks reflecting the presence of various polyphenols in MSE. Retention times 4.84, 4.94, 6.24, 6.39, 6.79, 7.28, 7.71, and 8.85 min. were detected for caffeic acid, sinapic acid, 3,4-dihydroxybenzoic acid, gallic acid, coumaric acid, quercetin, kaempferol, and ferulic acid, respectively.

We have previously shown potent antioxidative potential of *S. cumini* extracts indicating that *S. cumini* is rich in phenolic compounds. To evaluate this, we performed reverse-phase HPLC of *S. cumini* MSE which showed that indeed *S. cumini* MSE is enriched with a concoction of polyphenols, viz., corilagin, doxycycline, ellagic acid, ferulic acid, epigallocatechin gallate, gallic acid, kaempferol, quercetin, and sinapic acid (Figure 6). The structures, retention time, and quantity of these polyphenols are shown in Table S1. The presence of these polyphenols was validated using standard polyphenols in HPLC (Fig. S2). The protective effects of these polyphenols have been shown to be implicated in several diseases.

**Figure 7:**
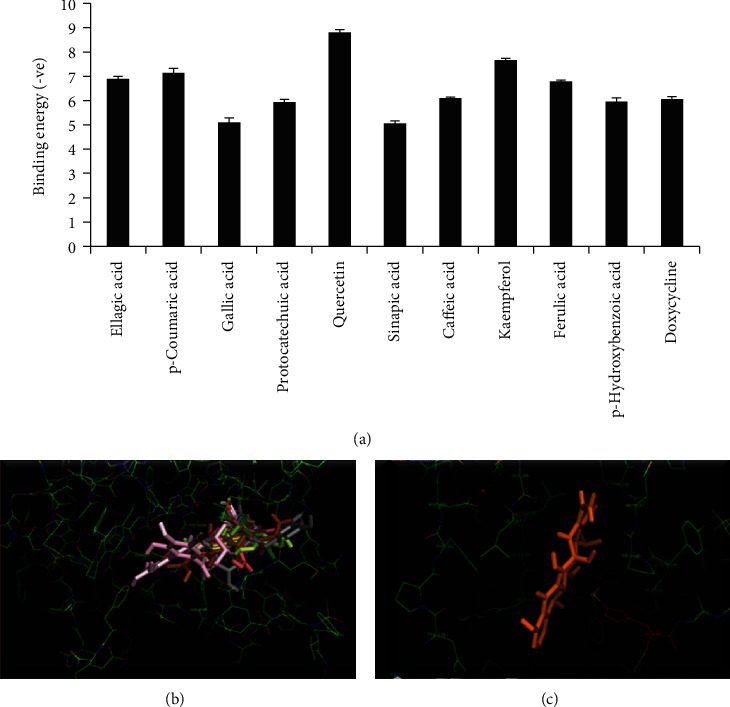
(a) Matrix metalloproteinase-9 (MMP-9) and polyphenol binding energy interaction graph. The *x*-axis represented various MSE polyphenols/doxycycline, and the *y*-axis showed (-ve) binding energy. (b) Predicted binding mode of polyphenols and doxycycline to MMP-9 (gelatinase-B): docked structures of gallic acid (pink), p-hydroxybenzoic acid (dark green), ellagic acid (blue), kaempferol (gray), p-coumaric acid (orange), protocatechuic acid (yellow), sinapic acid (light green), caffeic acid (brown), ferulic acid (mustard), and quercetin (red) with MMP-9. (c) Docking pose of doxycycline with MMP-9.

Our results showed that *S. cumini* MSE has a protective function under high-glucose conditions by inhibiting NF-*κ*B translocation and gelatinase-B expression as well as activity, but whether it had a direct influence on gelatinase-B activity remains unresolved. We therefore evaluated if *S. cumini* polyphenols have a tendency to bind to MMP-9. *In silico* molecular docking studies showed that these polyphenols have a binding tendency to MMP-9 with variable binding energies (Figures 7(a)–7(c)). Of these, quercetin had the maximum binding energy followed by kaempferol, ferulic acid, and caffeic acid. Interestingly, gallic acid showed the least binding energy explaining a better efficacy of *S. cumini* MSE than gallic acid in some of the results obtained above. Intriguingly, all the polyphenols were docked on the Zn^2+^ metal-binding site (consensus sequence HEBGHxLGLxHS), on three histidine residues (His226, His230, and His236) which belong to the substrate-binding site of MMP-9. These results show that despite the fact that *S. cumini* MSE regulates gelatinase-B expression via NF-*κ*B activity, the polyphenols within the MSE are capable of binding directly to gelatinase-B and could therefore have a direct effect on gelatinase-B activity.

## 4. Discussion

Diabetes and its long-term complications are a serious threat worldwide despite the availability of significant therapeutic options. Epidemiological and pathophysiological studies indicate that diabetes mellitus (DM) patients have increased risks of cardiovascular diseases. Even a slight increase in the hemoglobin A1c (HbA1c) level may lead to 20-30% upsurge in CVDs [40]. ECM dysfunction and alteration in MMP activity lead to the degradation of various structural proteins and several CVDs. MMP inhibitors have been shown to be effective in mediating efficient cardioprotection. However, due to the poor bioavailability, high toxicity, and failure of synthetic MMP inhibitors in clinical trials, attention has shifted to plant-derived MMP inhibitors [41].

The polyphenolic content-enriched functional food could have shown a promising approach towards the treatment of such diseases. Such natural product-derived drugs would have limited toxicity and side effects with better therapeutic indices [42]. The role of *S. cumini* seeds on HG-induced cardiac stress is not known so far; therefore, in the present study, methanol extract of *S. cumini* seeds was used to examine its effect on HG-induced cardiomyocytes. The effect was also examined with a purified form of a known constituent of *S. cumini* seed extract, gallic acid, a well-known cardioprotectant in diabetes-induced myocardial dysfunction and also an antioxidant [43]. We found *S. cumini* MSE to be enriched with polyphenols and had the potential to suppress HG-induced gelatinase-B activity in H9C2 cells which was comparatively better than that of gallic acid. The following major findings have emerged from this study: (i) *S. cumini* MSE phytoconstituents are cardioprotective against HG-induced changes in H9C2 cell morphology, (ii) *S. cumini* MSE polyphenols significantly suppress MMP-9 expression and activity in HG-induced H9C2 cells, and (iii) *S. cumini* MSE inhibits HG-induced nuclear localization of NF-*κ*B and overexpression of TNF-*α* and IL-6, (iv) HPLC identified that *S. cumini* polyphenols show competitive inhibition with Zn^2+^ ions for the metal-binding domain of gelatinase-B in molecular docking study. *S. cumini* polyphenols bind more efficiently than the currently available FDA-approved synthetic MMP inhibitor, doxycycline, indicating the potential use of *S. cumini* polyphenols as safe MMP inhibitors since *S. cumini* MSE alone did not cause any significant change in cellular viability and morphology.

The H9C2 cell line is an efficient in vitro model for studying cardiac stress as it emulates the responses similar to those observed in primary cardiomyocytes [44]. The concentration of high glucose used in this study reflects in vitro hyperglycemic diabetic stress [45].

Adult cardiac myocytes are terminally differentiated cell types which do not proliferate but execute various physiological functions. Under various stress conditions, cardiomyocytes enlarge and undergo hypertrophy, a compensatory cellular response of the heart to the imposed hemodynamic burden for an increased cardiac output [46]. The increase in cell size is a major hallmark of cardiac cell hypertrophy, and the agonists such as norepinephrine, isoproterenol, and glucose induce hypertrophy of cardiomyocytes, which cause reexpression of some fetal genes, for instance, atrial natriuretic factor (ANF), brain natriuretic peptide (BNP), and *β*-myosin heavy chain (*β*-MHC) [47].

We observed similar significant enlargement of cardiomyocytes upon exposure to HG, which was reversed by *S. cumini* MSE treatment suggesting potential cardioprotective effect of *S. cumini* against HG-induced stress. Enhanced activity of MMPs by high glucose may contribute to matrix reorganization, whereas marked reduction in MMP activity by MSE treatment showed its ability to reverse HG-induced changes in cardiomyocytes. *In situ* zymography, a relatively superior method that helps to map the activity within the cell, showed increased activity of MMPs that was prevented by *S. cumini* MSE. These data strongly support that MSE possesses strong MMP-inhibiting activity.

Gelatinase-A (MMP-2) is constitutively expressed in the heart while gelatinase-B is an inducible protease, and we conducted our further study with gelatinase-B due to its inducible response to various stimuli [48]. Indeed, elevated levels of gelatinase-B have been proposed as a serological cardiac stress marker as its expression increases markedly in diabetic cardiomyopathy [49]. Interestingly, we observed a mild induction of gelatinase-B expression on treatment with MSE extract alone. Since the MSE extract is a crude preparation, it may contain several other phytochemicals which may be responsible for this induction at baseline. Furthermore, the extract is protective under glucose-induced stress and behaves differently compared to baseline indicating its efficacy in disease conditions. Additionally, an increase in gelatinase-B expression may not be sufficient to create a functional effect, and we do not observe any effect of MSE extract on gelatinase-B activity at baseline.

To investigate the effect of *S. cumini* MSE on HG-induced gelatinase-B expression, we examined the activation and translocation of transcription factor NF-*κ*B. In fact, gelatinase-B promoter is highly conserved and contains an NF-*κ*B-binding site, and NF-*κ*B is known to upregulate the production of MMP-9 when induced by various proinflammatory cytokines [50]. The present study shows that HG induced the localization of NF-*κ*B from the cytoplasm to the nucleus indicating activation of gelatinase-B transcription. *S. cumini* MSE treatment regulated this alteration in nuclear localization of NF-*κ*B leading to decreased expression of gelatinase-B in HG-stimulated cardiomyocytes. Additionally, MSE also downregulated the expression of inflammatory markers such as TNF-*α* and IL-6 which were known to be critical in cardiomyopathy [51].

Thus, our study demonstrates that *S. cumini* MSE can be used as a potential functional food to suppress the expression of proinflammatory cytokines and gelatinase-B in HG-induced stress in cardiomyocytes. Although a clear mechanism is not yet known, the presence of hydroxyl/carbonyl groups on the phenolic rings of a number of MMP inhibitors has been suggested to chelate active Zn^2+^ ions and inhibit MMP activity [52].

*S. cumini* extracts prepared from different plant parts such as pulp, seed, leaves, and kernel have differential distribution of chemical constituents, especially the polyphenols, which are reported to possess a range of pharmacological potential. To know the status of polyphenols in MSE, a quantitative analysis was performed by HPLC, which indicated the presence of many polyphenols in MSE. The major polyphenols found in MSE (in 100 g of seed powder) were p-coumaric acid (71.9 ± 0.05 mg), gallic acid (53.0 ± 0.06 mg), protocatechuic acid (40.6 ± 0.10 mg), quercetin (29.6 ± 0.05 mg), sinapic acid (30.8 ± 0.14 mg), caffeic acid (15.8 ± 0.03 mg), kaempferol (10.2 ± 0.09 mg), ellagic acid (5.8 ± 0.09 mg), ferulic acid (2.71 ± 0.02 mg), and p-hydroxybenzoic acid (0.52 ± 0.04 mg) (Table S1).

The polyphenolic compounds that were identified and quantified in our study have been shown to exhibit cardioprotective potential as identified by various research groups using *in vitro* and models. p-Coumaric acid attenuated apoptosis in isoproterenol-induced myocardial infarction by inhibiting oxidative stress [53]. Gallic acid, a known cardioprotectant, has been found to suppress the expression of cardiac troponin-T, a cardiac arrest marker enzyme, lipid peroxidation products, and antioxidative enzymes [54].

Quercetin was reported to prevent endothelial dysfunction and decrease blood pressure, oxidative stress, and end-organ damage in hypertensive animals [55]. Derivatives of benzoic acid have been reported to activate Nrf2 signaling in the heart leading to overexpression of antioxidant enzymes, thereby decreasing oxidative stress and associated problems such as endothelial dysfunction and atherosclerosis [56].

Sinapic acid prevented ischemia/reperfusion, cardiac hypertension, and remodeling [57]. Caffeic acid derivatives exerted a protective effect during streptozotocin- and isoproterenol-induced cardiac stress [58]. Kaempferol has been reported to be cardioprotective by regulating the membrane-bound ATPases in streptozotocin-induced diabetic rats [59]. Ferulic acid is also found to suppress anticancer drug-induced cardiotoxicity [60]. Ellagic acid improved arrhythmic condition by upregulating endothelial nitric oxide synthase (eNOS) and alleviating oxidative stress [61]. These evidences strengthen and support our hypothesis that MSE, which is a mix of these polyphenolics, could be a potential cardioprotective agent in HG-induced stress.

The mechanism of MMP-mediated cardioprotection by these compounds is not yet known. To understand this further, the polyphenols identified in HPLC were used to study interaction with the MMP-9 active site. Our docking studies revealed good binding energies of HPLC-identified molecules with MMP-9. Such ligand-protein interaction was based upon the inhibitor/substrate binding site, suggesting that MMP-9 inhibition by *S. cumini* MSE was due to polyphenols present in MSE that act as competitive inhibitors with the substrate. The results of the docking study also explained that there was a direct interaction between gelatinase-B and the polyphenolic components of *S. cumini* MSE, which hindered its activity by targeting critical histidine residues of the metal-binding domain, thereby preventing the binding of Zn^2+^ to the active site which in turn could inhibit MMP activity. The docking results suggested that *S. cumini* polyphenols especially quercetin, kaempferol, caffeic acid, and ferulic acid could be efficient MMP-9 inhibitors and thereby may be potential suppressors of HG-induced cardiac stress.

Structure-activity relationship (SAR) was established on the basis of results obtained in docking studies which revealed that the two structural elements, namely, chromone ring and hydroxyl groups in the aromatic ring in the polyphenols of MSE, were the governing factors for the activity. Chromone core-bearing polyphenols such as quercetin and kaempferol showed excellent binding affinity with MMP-9 as compared to doxycycline. It also appeared that along with chromone moiety, the number and position of hydroxyl groups also played a critical role in deciding the MMP inhibitory activity of polyphenols. Other MSE polyphenols also displayed satisfactory binding affinity with MMP-9 possibly due to the presence of hydroxyl groups.

It was also observed that polyphenols having the hydroxyl group attached directly to the benzene ring had more binding affinity (p-hydroxybenzoic and protocatechuic acid) as compared to those having hydroxyl groups in the side chains (sinapic acid). The following order of affinity has been observed in docking analysis: quercetin > kaempferol > ferulic acid > caffeic acid/p‐coumaric acid > p‐hydroxybenzoic acid/protocatechuic acid > sinapic acid > gallic acid. Our docking studies, therefore, correlated very well with SAR wherein maximum binding affinity of quercetin with MMP-9 was observed due to the presence of chromonering and five hydroxyl groups responsible for its excellent affinity with MMP-9. Henceforth, the docking and SAR data suggested that *S. cumini* MSE polyphenols may act as potent MMP-9 inhibitors.

The greatest hurdle in using herbal supplements as potential drugs has been that researchers try to identify a single bioactive molecule to modify certain pathological parameters, whereas practically the crude extracts containing many bioactive components show better pharmacodynamic synergistic potency [62]. The crude extracts could be more helpful as they are derived from natural plant parts which are usually biocompatible with the human system and thus would have low toxicity and higher bioavailability [63]. Our results also supported the concept of collaborative interaction of phytochemical-enriched *S. cumini* extract that could act in synergy for effective cardioprotection under high-glucose stress.

The polyphenol components of *S. cumini* MSE such as gallic acid alone are not used as dietary supplements as they inhibit the food intake as well as do not fulfill the criteria of effective oral dosing. Pharmacokinetic studies have shown that blood levels having 10^−6^ M concentration of gallic acid restrict the appropriate functioning of the transport system [64]. Therefore, proposing the potential use of such a plant extract as *S. cumini* as a MMP inhibitor would be advantageous in terms of its cost, availability, and safety.

In this regard, use of *S. cumini* as a nutritional supplement or functional food for diabetics prove to be a potent natural suppresser for gelatinase-B-mediated stress in HG-induced condition. Based on the data of the present study, a model can be proposed to understand the effect of *S. cumini* as a MMP inhibitor and a cardiac stress reliever via targeting the molecules at multiple levels. The study has significant relevance for the society as most of the identified MMP inhibitors possess severe side effects like musculoskeletal syndrome (MSS). Doxycycline is the only FDA-approved MMP inhibitor in the market currently that is only for periodontal diseases.

Our results show the strong interaction of phytochemicals with gelatinase-B that may act in synergy for effective cardioprotection under hyperglycemic stress. In this regard, the usage of *S. cumini* as functional food proved to be a potent natural suppresser for gelatinase-B-mediated HG-induced stress. A significant relationship between ECM integrity in HG-induced stress and the content of phenolic components of MSE supported this perception. Taken together, the present results suggest the potential of *S. cumini* MSE as an herbal-based therapy for the treatment of HG-induced cardiac stress. Due to the overwhelming potentials of *S. cumini* polyphenols, further in-depth analysis of the extract and validation on appropriate animal models are required to understand the precise mechanism for designing specific therapies against ECM remodeling in diabetic cardiomyopathy.

Myocardial remodeling could be categorized as either adaptive or pathological. Initial changes in the myocardium may appear phenotypically similar in both cases, but the physiological effects of each are drastically different. Adaptive myocardial remodeling occurs due to high stress on the vascular walls or increased workload. It helps to counter the stress and restore normal ventricular function. Observations in the myocardium of high functioning athletes show changes in Left Ventricular (LV) geometry allowing improved compliance, enhanced filling through LV dilation, and better stroke volume [65]. On the other hand, in pathological remodeling, the LV chamber dilation leads to diminished compliance and significantly lower forward stroke volume [66]. MMP-2 has a crucial role in the progression of cardiac remodeling in response to pressure overload. An increase in MMP-2 activity can lead to fibrosis in LV hypertrophy, which may be due to direct proteolysis of cardiac ECM components, as well as by generating a profibrotic response, which further results in adaptive remodeling [67]. MMP-9 is a critical marker of LV remodeling; a higher MMP-9 level indicates more extensive adaptive LV remodeling [68]. It is also observed that in the patients having LV hypertrophy and with a history of hypertension, plasma levels of MMP-2 were significantly enhanced as compared to control subjects [67]. However, MMP-9 activates various chemokines, such as CXCL5, CXCL6, and CXCL8, releases cell surface receptors (e.g., tumor necrosis factor-*α* receptor), and eventually contributes to pathological remodeling. It has several other inflammatory mediators such as activator protein-1, specificity protein-1, and NF-*κ*B sites that make it a possible target for myocardial remodeling in atherosclerosis and heart failure [69].

## 5. Conclusion

In conclusion, the present study on the one hand highlights the role of MMPs, especially gelatinase-B, in HG-induced cardiomyopathy in rat heart-derived H9C2 cardiomyoblast cells, and on the other hand, it elaborates the role of *S. cumini* MSE in suppressing HG-stimulated gelatinase-B expression and activity in cardiomyocytes. Further, strong binding energy of interaction of *S. cumini* polyphenols and gelatinase-B protein suggests close interaction of the polyphenols with gelatinase-B which could be responsible for its inhibition. Taken together, being a potent source of polyphenols with MMP inhibition potential, *S. cumini* may be useful as a functional food and dietary supplement in HG-induced and gelatinase-B-mediated cardiac stress and cardiomyopathy. This study will certainly enhance the scientific opportunities in deriving the novel inhibitors from functional foods in developing countries.

## Data Availability

Data will be available on request.

## Supplementary Materials

Supplementary 1 Fig. S1: schematics of the methodology adopted to study the protective effect of *S. cumini* methanol seed extract (MSE) on high-glucose- (HG-) induced cardiac stress.

Supplementary 2 Fig. S2: HPLC chromatogram: (A) gallic acid and p-coumaric acid, (B) quercetin and ellagic acid, (C) kaempferol and ferulic acid, (D) protocatechuic acid and sinapic acid, and (E) caffeic acid and p-hydroxybenzoic acid.

Supplementary 3 Table S1: HPLC analysis of methanol seed extract of *S. cumini*.
