# Supplementary figures and images for: Anti-inflammatory Effects of S. cumini Seed Extract on Gelatinase-B (MMP-9) Regulation against Hyperglycemic Cardiomyocyte Stress
Source: Oxid Med Cell Longev. 2021 Mar 3;2021:8839479. doi: 10.1155/2021/8839479 (PMC7953863; doi:10.1155/2021/8839479)

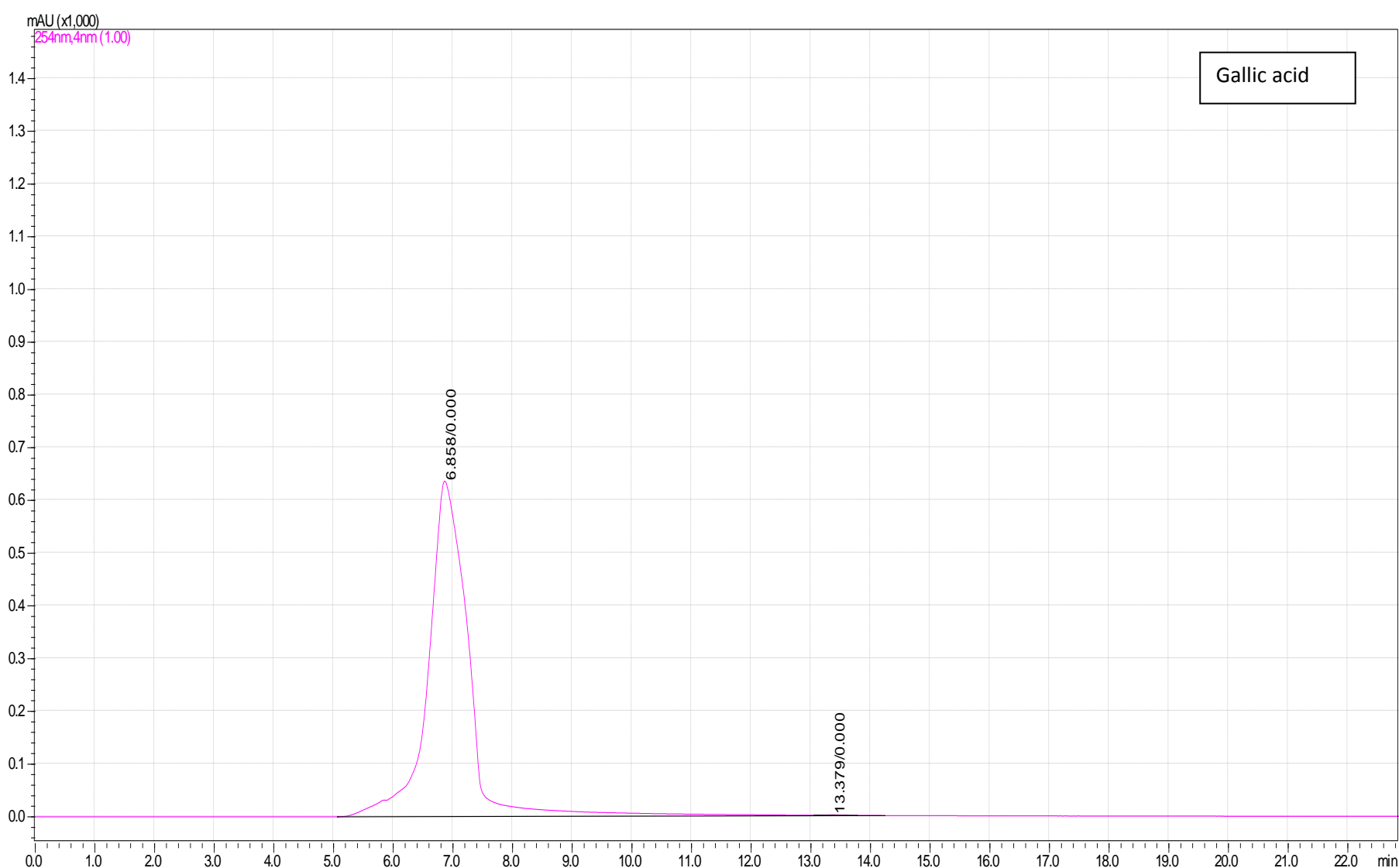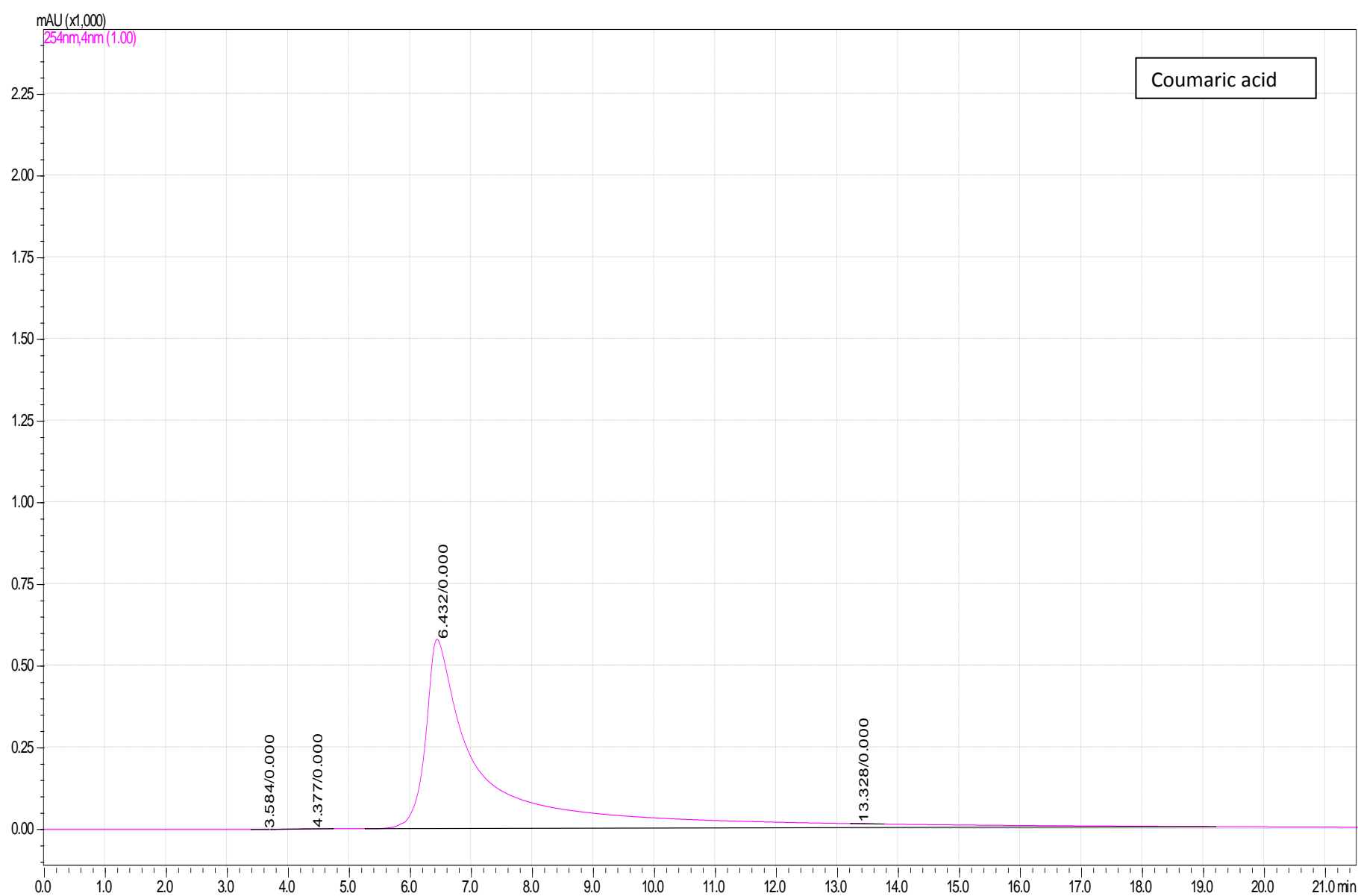

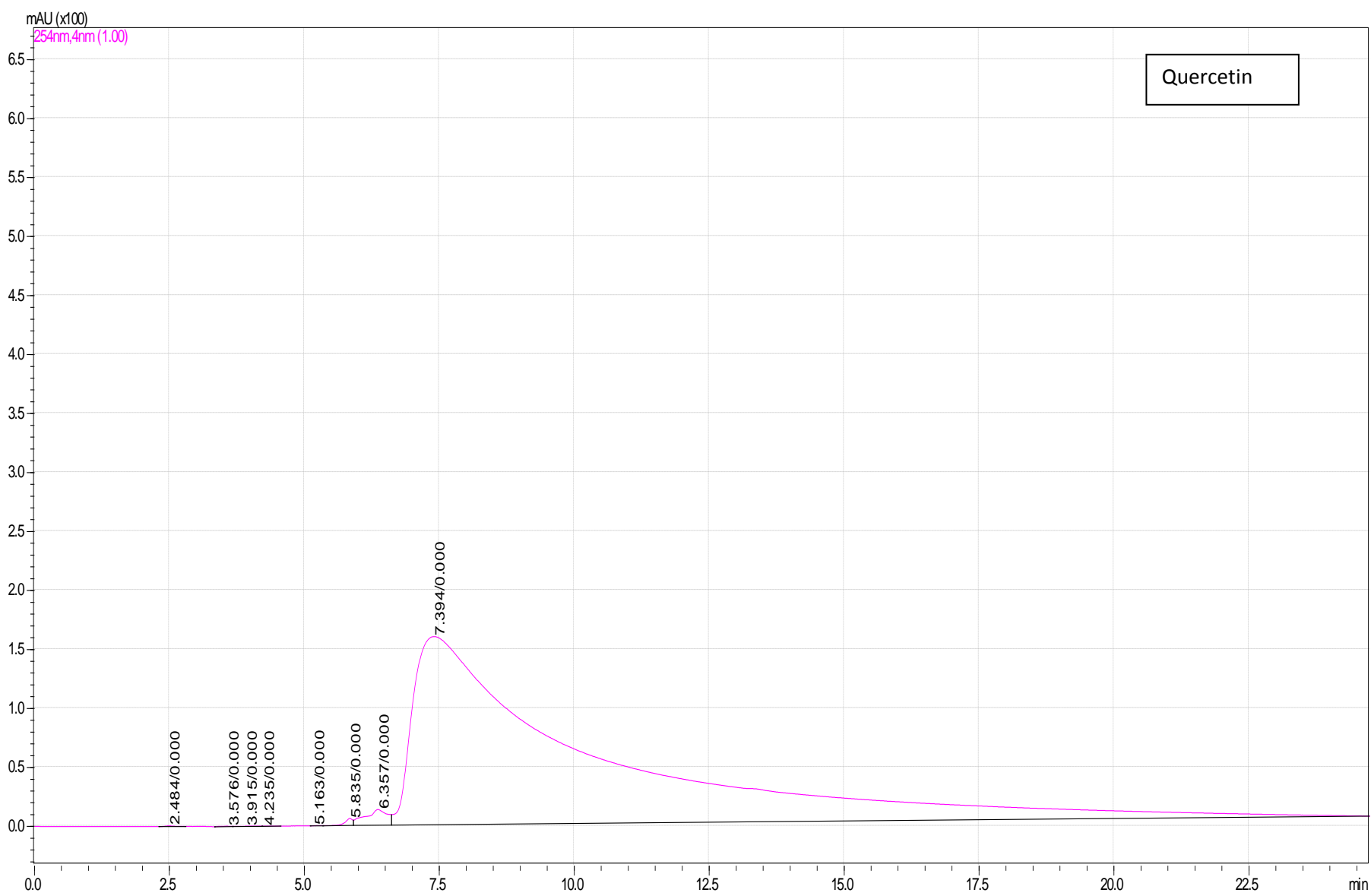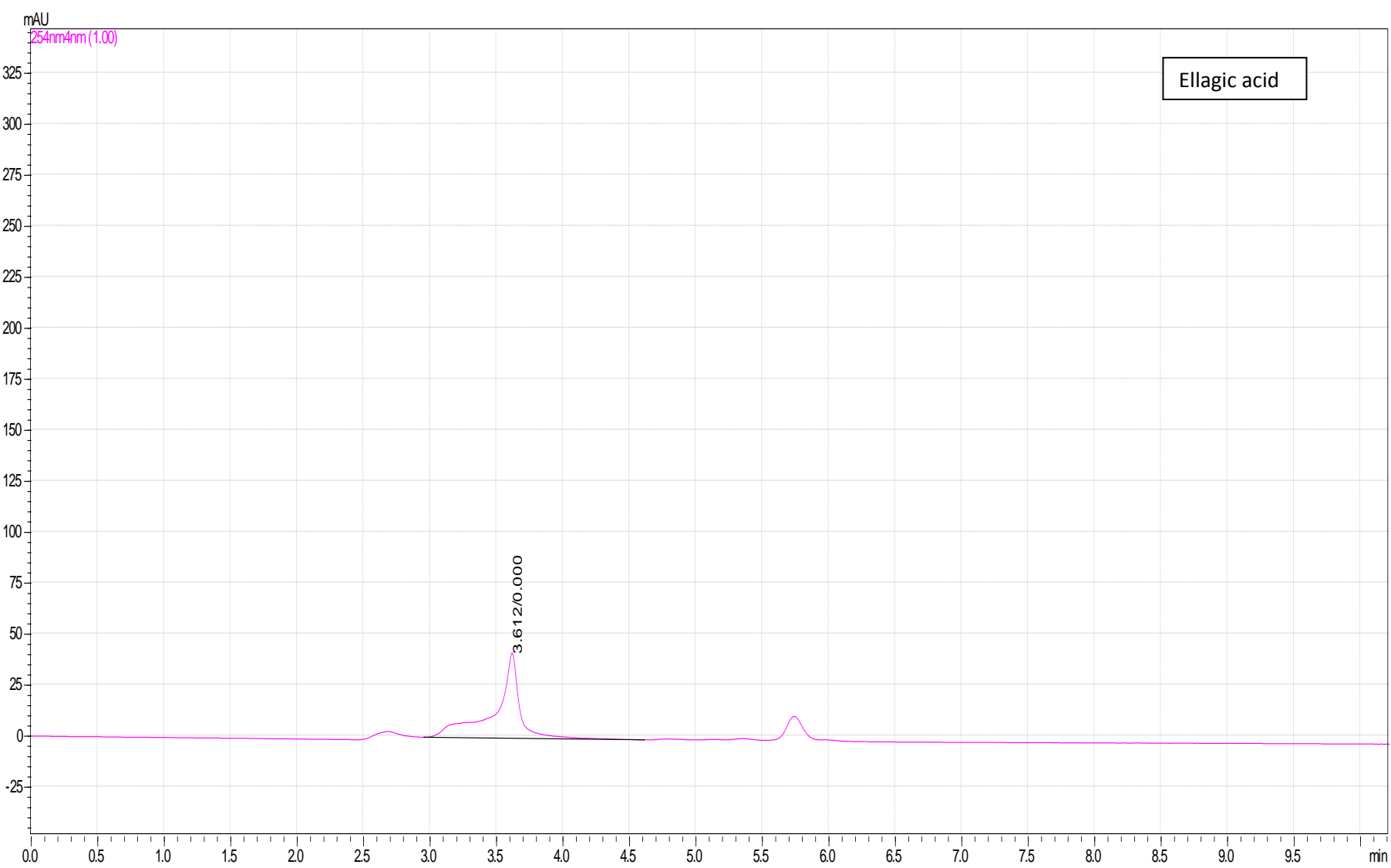

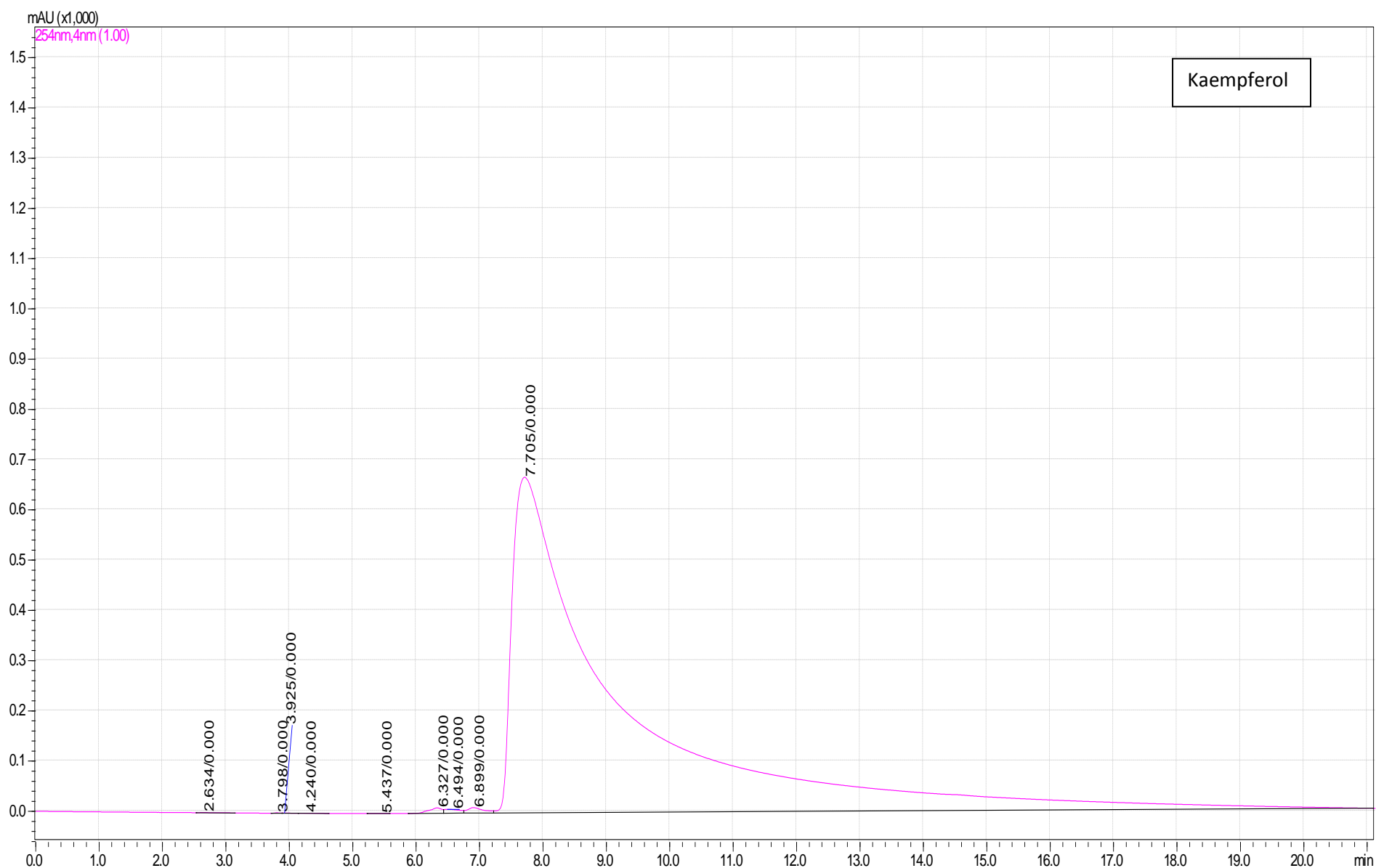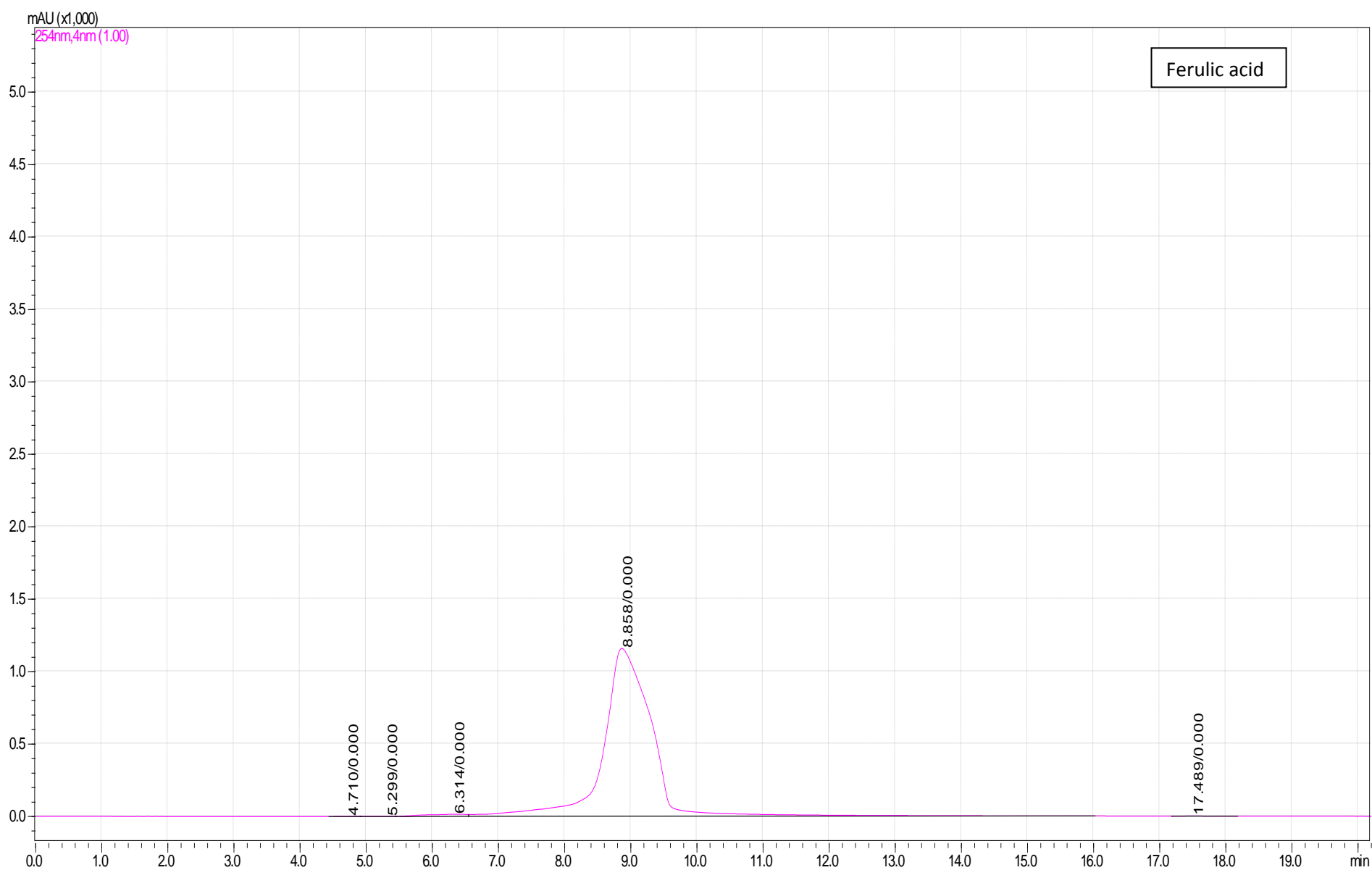

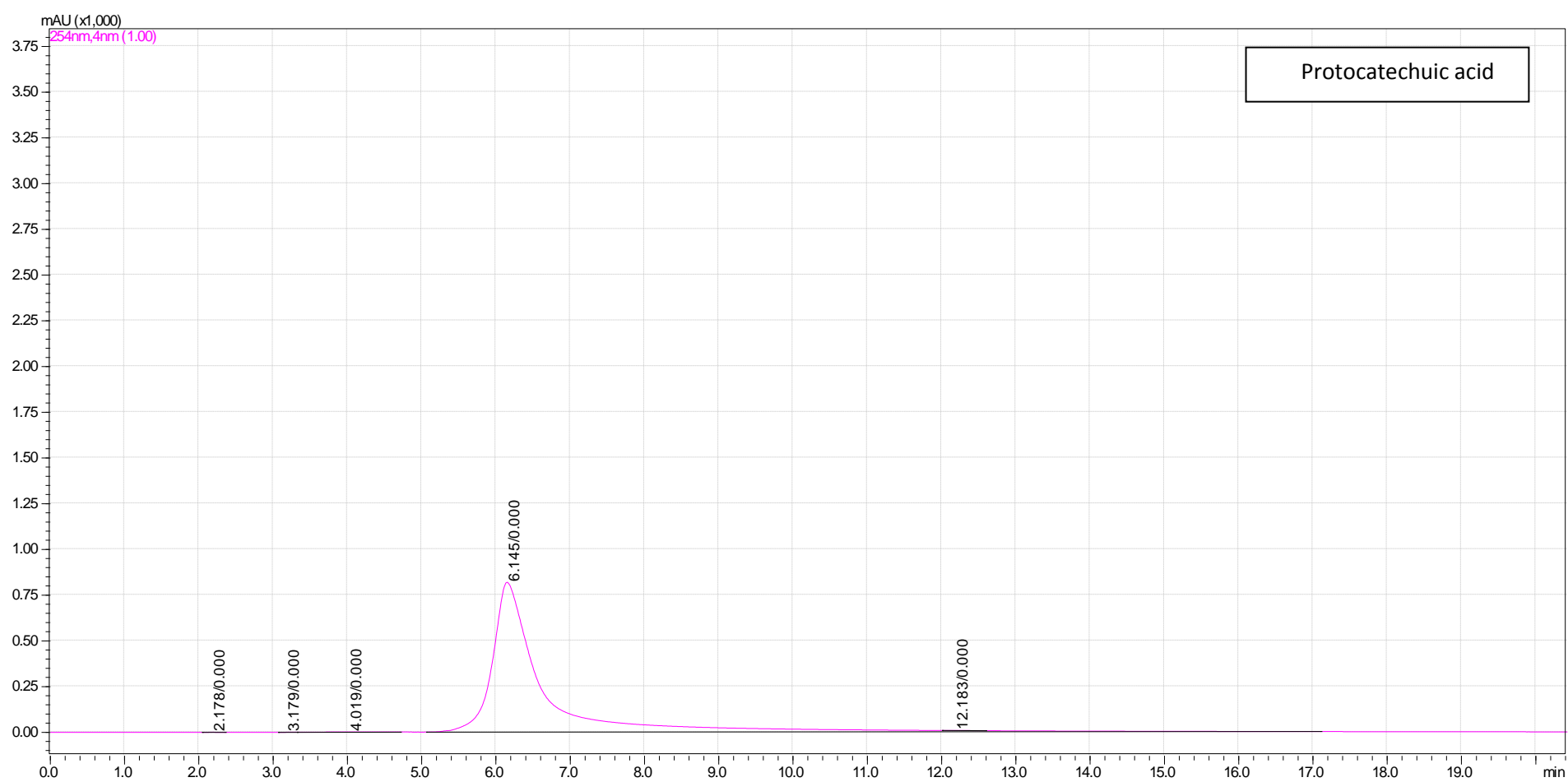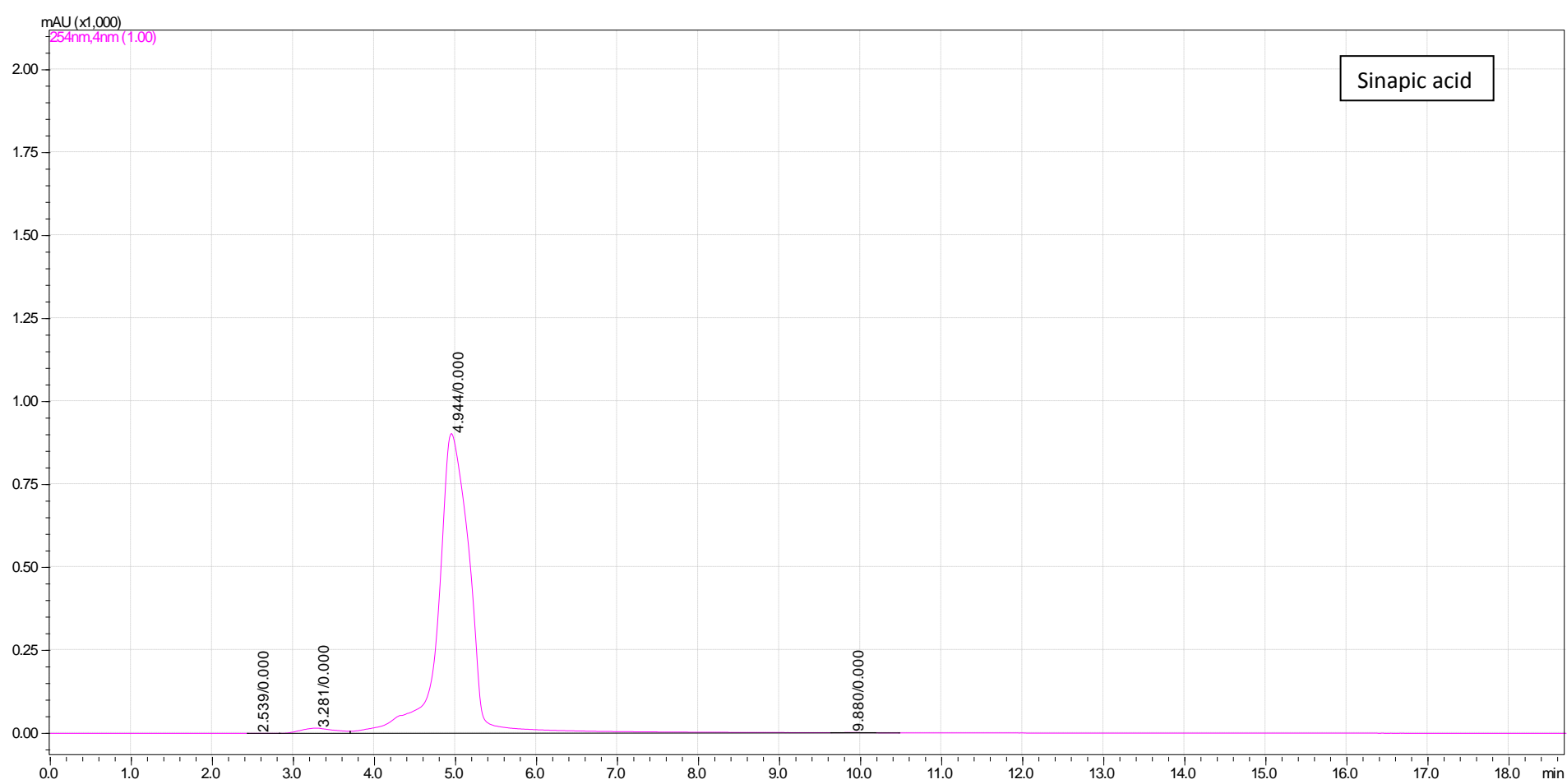

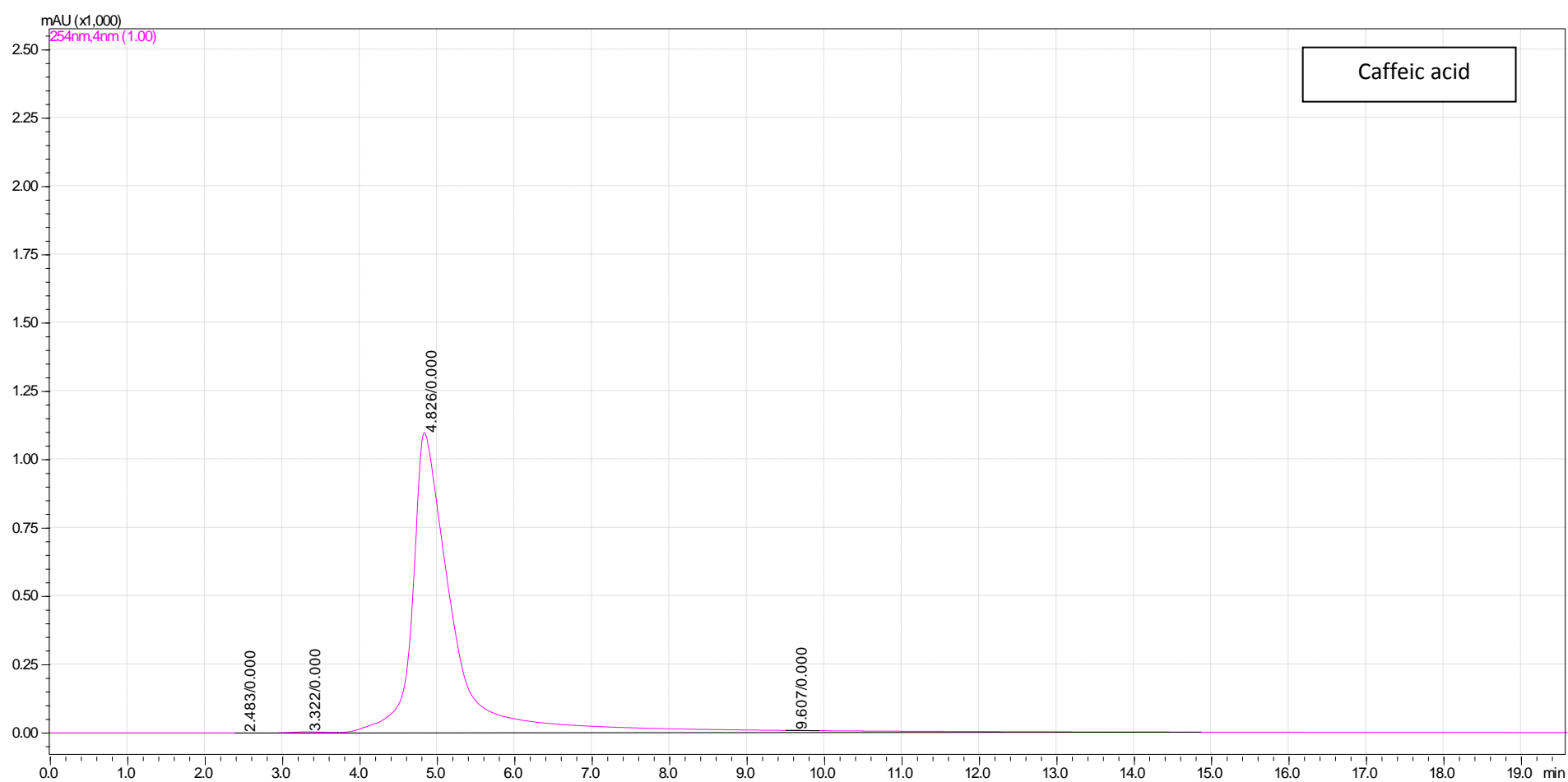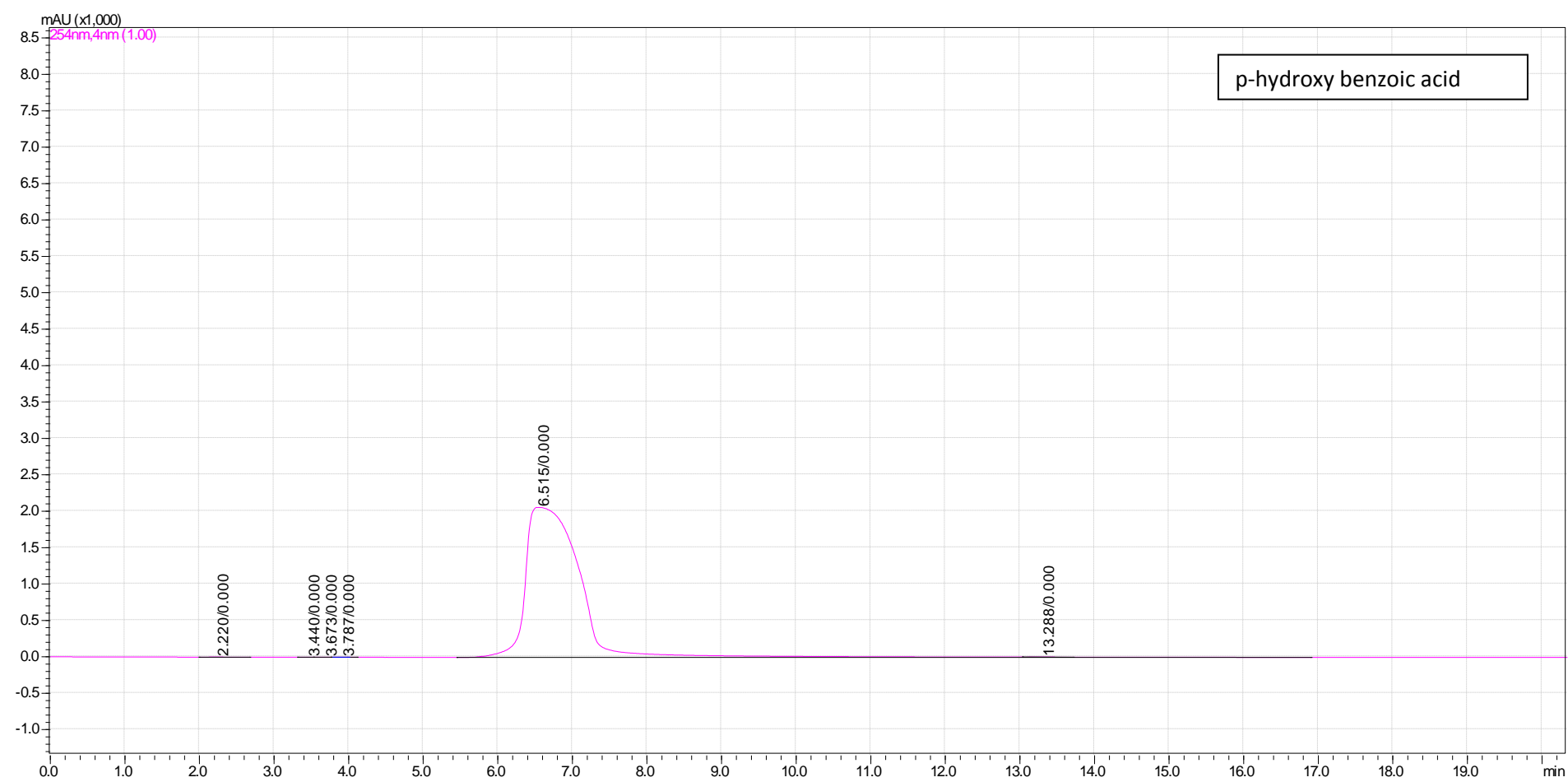

Supplement: Supplementary 1 — Fig. S1: schematics of the methodology adopted to study the protective effect of S. cumini methanol seed extract (MSE) on high-glucose- (HG-) induced cardiac stress. [file 8839479.f1.pdf]

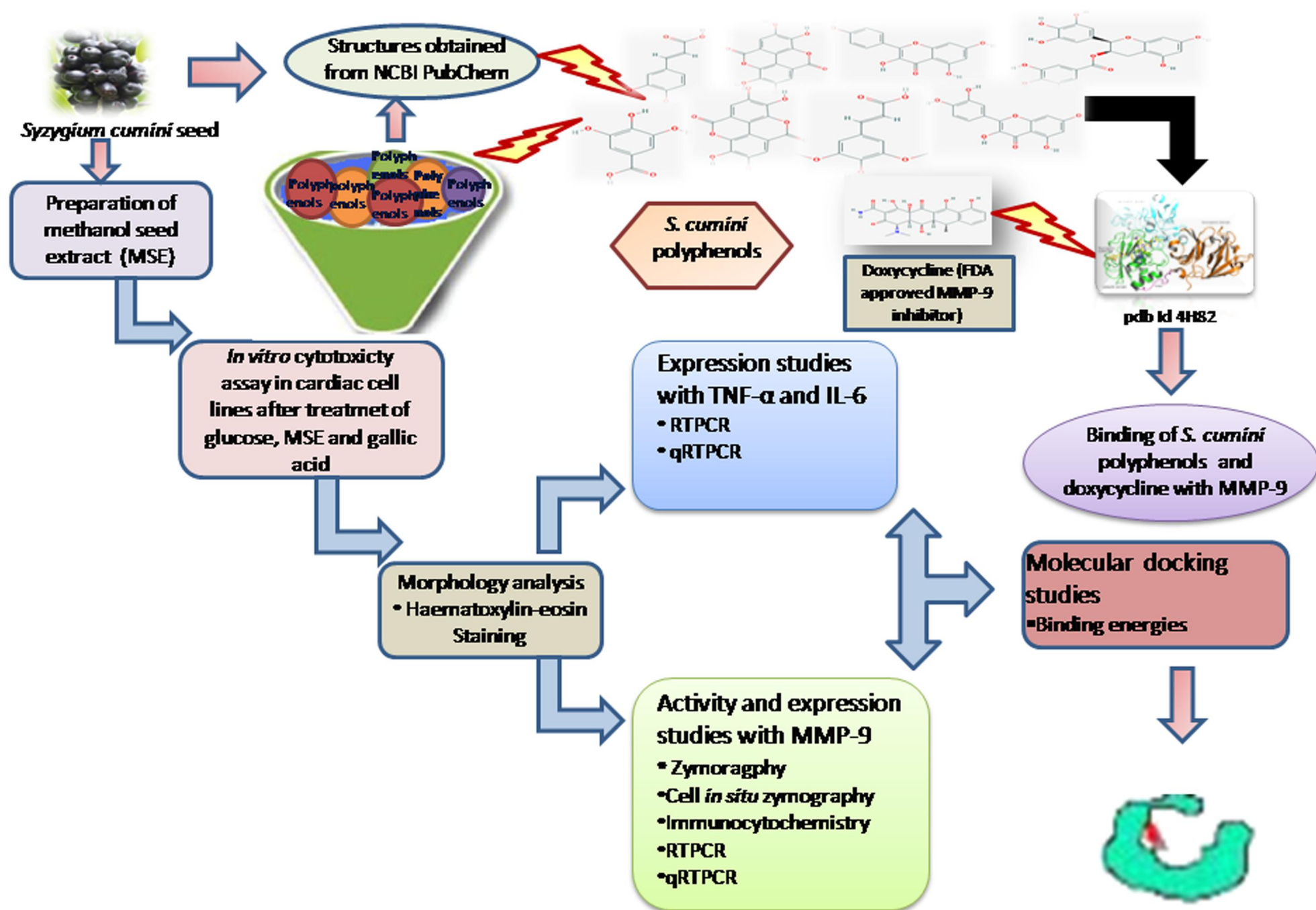

Supplement: Supplementary 2 — Fig. S2: HPLC chromatogram: (A) gallic acid and p-coumaric acid, (B) quercetin and ellagic acid, (C) kaempferol and ferulic acid, (D) protocatechuic acid and sinapic acid, and (E) caffeic acid and p-hydroxybenzoic acid. [file 8839479.f2.pdf]
