# Supplementary material for: Anti-inflammatory Effects of S. cumini Seed Extract on Gelatinase-B (MMP-9) Regulation against Hyperglycemic Cardiomyocyte Stress
Source: Oxid Med Cell Longev. 2021 Mar 3;2021:8839479. doi: 10.1155/2021/8839479 (PMC7953863; doi:10.1155/2021/8839479)
Supplement: Supplementary 3 — Table S1: HPLC analysis of methanol seed extract of S. cumini. [file 8839479.f3.pdf]

S. No	Compounds identified and their Structure	Retention time	Quantity (mg/100g of dry weight)
1.	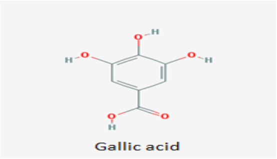	6.398	53.0±0.06
2.	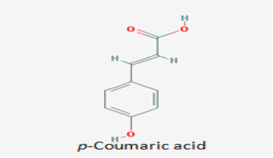	6.793	71.9±0.05
3.	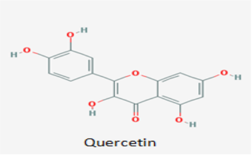	7.281	29.6±0.05
4.	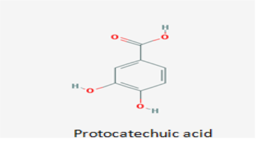	6.247	40.6±0.10
5.	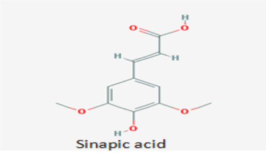	4.949	30.8±0.14
6.	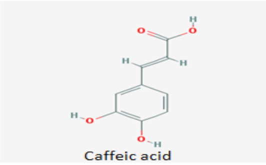	4.843	15.8±0.03
7.	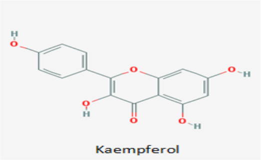	7.712	10.2±0.09
8.	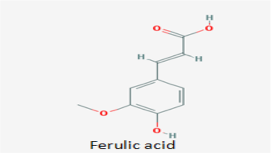	8.859	2.71±0.02
9.	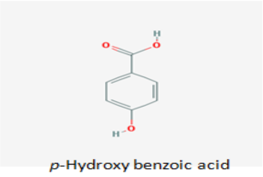	6.688	0.52±0.04

10.	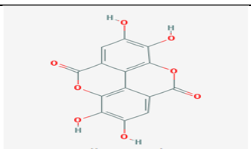 Ellagic acid	3.894	5.8±0.09
